# A potential explanation of the causal relationship between atherosclerosis and lung cancer from an immunological perspective: A Mendelian randomization and transcriptomics study

**DOI:** 10.1097/MD.0000000000047757

**Published:** 2026-02-20

**Authors:** Peinan Zhang, Xiaozhang Bao, Xike Wu, Yuheng Yang, Yanqi Sun, Ying Qian, Nan Tian

**Affiliations:** a College of Life Science, Zhejiang Chinese Medical University, Hangzhou, China.

**Keywords:** atherosclerosis, lung cancer, Mendelian randomization, transcriptomics, tumor microenvironment

## Abstract

The relationship between atherosclerosis (AS) and lung cancer has garnered growing interest recently, as their frequent co-occurrence jointly drives global mortality and worsens prognosis. Current observational evidence is inconclusive and remains vulnerable to residual confounding and reverse causality, underscoring the imperative for methodologically rigorous causal inference. The potential causal relationship between AS and lung cancer was investigated using Mendelian randomization (MR). Subsequently, transcriptomic analysis was conducted using public datasets, followed by the construction of an AS-associated lung cancer prognostic model using LASSO-Cox regression and the evaluation of its performance. Finally, ESTIMATE and ssGSEA algorithms were used to evaluate the 2 groups of immune infiltration. A significant inverse causal association between AS and lung cancer was demonstrated by MR analysis (*P* = .01, OR = 0.896, CI = 0.825 − 0.974). Furthermore, 2 risk groups of patients with lung cancer, characterized by different prognoses and immune landscapes, were stratified using a risk scoring model that comprised 3 AS-related genes (CD52, FABP5, and FCGR3A). The tumor microenvironment in the low-risk group of lung cancer had a higher proportion of immune cells, and the infiltration levels of neutrophils and mast cells were significantly higher than those in high-risk patients. The MR analysis in this study revealed that genetic alterations in AS were significantly associated with a reduced risk of lung cancer. Transcriptomic data indicated that chronic inflammation linked AS and lung cancer: inflammatory mediators drove AS yet restrained lung cancer progression in the tumor microenvironment, while AS-derived immune molecules and pathways further suppressed tumor growth.

## 1. Introduction

Atherosclerosis (AS) is a chronic inflammatory condition induced by various causes, including low-density lipoprotein, tobacco use, hypertension, and others. As atherosclerotic plaques progress, they can give rise to severe clinical outcomes, such as acute cardiovascular diseases and strokes.^[1,2]^ Cardiovascular diseases related to atherosclerosis are among the leading causes of mortality and morbidity worldwide.^[3]^ In recent years, numerous shared risk factors between AS and cancer have been identified, including obesity, diabetes, hypertension, smoking, and inflammatory status.^[4–6]^ Lung cancer ranks first among malignant tumors owing to its high incidence and mortality. Statistics indicate that there were about 2.2 million new cases of lung cancer in 2020. There were also approximately 1.8 million lung cancer-related deaths.^[7]^ The significant prevalence of AS and lung cancer in the general population has led to the identification of a reciprocal relationship between the 2 conditions in several observational studies.^[8–12]^ Specifically, research indicates that female patients with atherosclerosis face a significantly elevated risk of developing lung cancer compared to those without atherosclerosis (RR = 3.26, 95% CI: 1.95–5.46).^[8]^ Meanwhile, studies have also found that AS significantly suppresses the progression of lung cancer in mouse models.^[10]^ Notably, lung cancer can also significantly elevate the risk of AS (HR = 2.19, 95% CI: 1.82–2.59).^[13]^ Possibly due to the influence of numerous underlying confounding factors, the conclusions drawn from these observational studies are inconsistent. In addition, the epidemiological manifestations of AS and lung cancer may exhibit variability among diverse ethnic groups.^[14,15]^ Therefore, more refined methods need to be designed to ascertain the causal relationship between AS and lung cancer.

In comparison with conventional observational studies, Mendelian randomization (MR) effectively overcomes common limitations such as confounding bias, measurement error, and reverse causality, thereby providing more reliable causal inference.^[16]^ Therefore, the objective of this study is to infer the causal relationship between atherosclerosis and lung cancer through MR analysis. Furthermore, given the documented increase in mortality among lung cancer patients with concomitant atherosclerosis, we sought to elucidate the potential mechanisms underlying their association via transcriptomics.^[12]^ Additionally, a lung cancer prediction model is constructed based on atherosclerosis-associated genes to assess the prognosis of patients with both atherosclerosis and lung cancer.

## 2. Materials and methods

**Figure 1.**
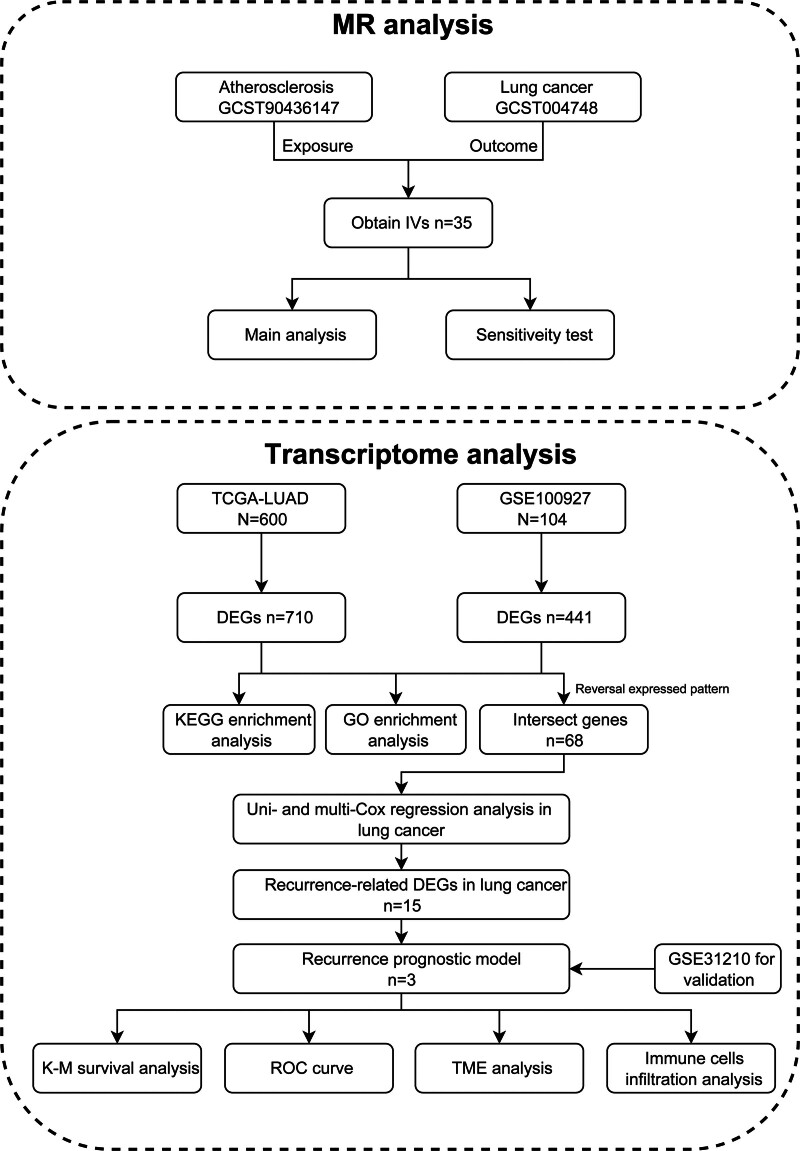
The flowchart for this study, where the main analysis section of MR analysis includes 5 methods: weighted median, MR-Egger, IVW, simple mode, and weighted mode. MR = Mendelian randomization, IVs = instrumental variables, IVW = inverse-variance weighted, TCGA-LUAD = the cancer genome atlas-lung adenocarcinoma, DEGs = differentially expressed genes, KEGG = Kyoto encyclopedia of genes and genomes, GO = gene ontology, K–M = Kaplan–Meier, ROC = receiver operating characteristic, TME = tumor microenvironment.

The overview design of our work is illustrated in Figure 1.

### 2.1. Data sources

All data for this MR analysis were downloaded from the GWAS Catalog database (https://www.ebi.ac.uk/gwas/). Summary statistical data for the exposure, AS, were sourced from dataset GCST90436147 (including 1324 cases and 400,595 controls of European ancestry).^[17]^ Data for the outcome, lung cancer, were derived from dataset GCST004748 (containing 29,266 cases and 56,450 controls of European ancestry).^[18]^ The study was dependent on publicly available summary-level data, and all original studies had received ethical approval.

### 2.2. Instrumental variable selection

MR analysis was premised on 3 core assumptions.^[16]^ Initially, single-nucleotide polymorphisms (SNPs) strongly associated with AS were chosen from the collected GWAS summary statistics according to a genome-wide significance threshold (*P *< 5 × 10^−8^). Next, linkage disequilibrium (LD) clumping was performed to ensure the independence of the instrumental variables (IVs). Specifically, SNPs with an *R*^2^ <0.001 within a 10,000 kb window were retained. Finally, the *F*-values were calculated to identify and exclude weak instruments. SNPs with *F*-values below 10 were removed.

### 2.3. MR analysis

Five MR methods were applied by the “TwoSampleMR” R package, including weighted median, MR-Egger, inverse-variance weighted (IVW), simple mode, and weighted mode. The IVW method was the primary approach for evaluating causal effects. Heterogeneity was assessed using Cochran *Q* test. The MR-PRESSO test was employed to both identify and remove the potential horizontal pleiotropy of SNPs (*P *<.05), thereby evaluating its influence on causal estimates. In addition, the MR-Egger intercept test was conducted for the purpose of examining directional pleiotropy. A leave-one-out sensitivity analysis was further performed to verify the robustness of the causal associations.

### 2.4. Transcriptomic analysis

#### 2.4.1. *Data acquisition*:

Microarray expression profiles and clinical data from GSE100927 (69 atherosclerosis samples and 35 controls) were downloaded from the Gene Expression Omnibus (GEO) database (https://www.ncbi.nlm.nih.gov/geo/). Information about RNA sequencing and clinical data that matched with patients (a total of 767 lung cancer specimens and 79 normal tissue specimens are included) was obtained from the GEO database (accession: GSE31210) and the Cancer Genome Atlas (TCGA) (https://portal.gdc.cancer.gov/). The TCGA cohort was used for training, and GSE31210 served as the validation set.

#### 2.4.2. Identification of overlapping differentially expressed genes:

Differentially expressed genes (DEGs) in AS or lung cancer compared with respective normal tissues were screened via the “limma” R package after data normalization. The filtering criteria for AS were adjusted to *P*-value <.05 and |log_2_FC| >1.0. Meanwhile, for lung cancer, the thresholds were set as adjusted *P*-value <.05 and |log_2_FC| >1.5.

#### 2.4.3. Functional enrichment analysis:

To further examine the biological mechanisms underlying the DEGs, KEGG and GO enrichment analysis were performed to uncover significantly enriched pathways. The limit for significance was an adjusted *P*-value of <.05. We used GSEA enrichment analysis to find the main pathways that are involved in the development of both AS and lung cancer. Through intersection analysis, we found the considerably enriched KEGG pathways that were shared across the 2 disorders and will look into them further.

#### 2.4.4. Construction and validation of the lung cancer-associated model

Based on the results from MR analysis and Gene Set Enrichment Analysis (GSEA), we selected overlapping DEGs exhibiting opposite expression patterns. Specifically, among the overlapping DEGs, we identified those showing a positive expression pattern in AS and a negative expression pattern in lung cancer. First, the RFS-associated DEGs of lung cancer patients were identified via LASSO regression in the “glmnet” package. A prognostic model comprising 3 genes was constructed. The risk scores were defined by the following formula: $\underset{x=1}{\overset{n}{\sum }}\;coefx*expx$, where the *coef* represents the coefficient value, and *exp* means the gene expression level of each gene. Based on the optimal risk cutoff, lung cancer patients were divided into low- and high-risk groups.

#### 2.4.5. Prognostic analysis

Subsequently, differences in overall survival (OS) and recurrence-free survival (RFS) were analyzed using the “survival” R package. Uni- and multi-Cox regression analyses were carried out on selected clinical features and the risk score to determine the independent prognostic role of each variable. The prognostic capability of the risk score was assessed using receiver operating characteristic (ROC) analysis and the area under the curve. Furthermore, the GSE31210 dataset, comprising 20 normal samples and 226 LUAD samples, was employed as a validation set to verify the predictive capability of the risk score. The risk score for the GSE31210 cohort was calculated using the same formula derived from the TCGA cohort, and patients were categorized into low and high groups based on the optimal cutoff value.

#### 2.4.6. Tumor microenvironment and immune cells infiltration analysis

To comprehensively evaluate the TME, enrichment scores calculated via the single-sample GSEA (ssGSEA) algorithm by the “GSVA” R package were analyzed to evaluate the abundance of 29 types of human TME-infiltrating cells. The ESTIMATE algorithm was used to compute stromal score, immune score, and tumor purity.

#### 2.4.7. Statistical analysis:

All statistical analyses were performed using R software (version 4.4.3). Two-tailed t-tests were used throughout this study; for data that were non-normally distributed, the Wilcoxon test was applied. The significance thresholds were *P* <.05, *P <.05, **P <.01, *** *P* <.001 for all statistical tests.

## 3. Results

### 3.1. Causal relationship in AS and lung cancer

**Figure 2.**
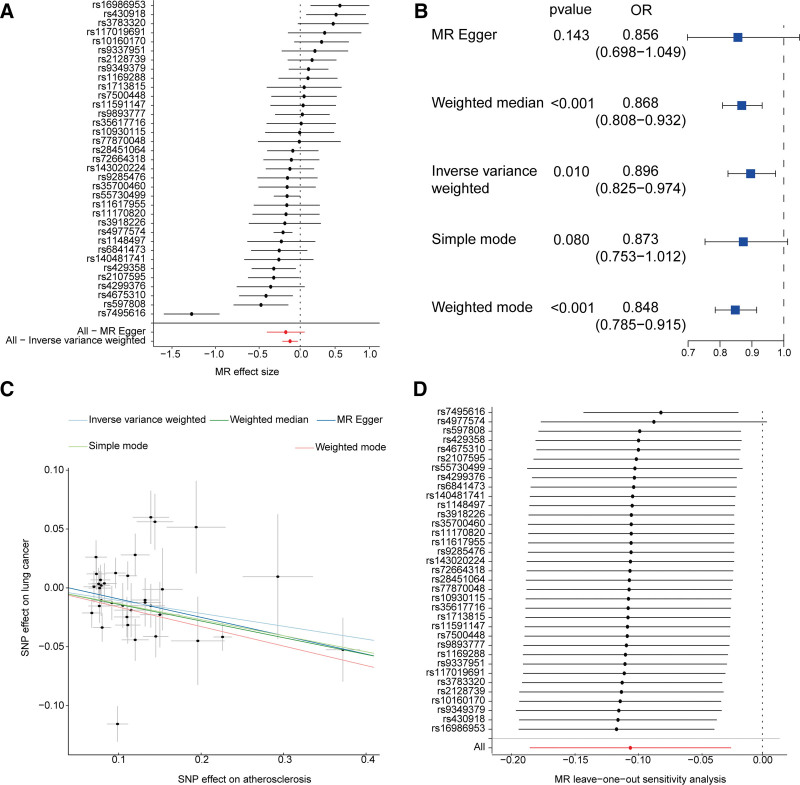
The causal relationship between atherosclerosis and lung cancer. (A) Forest plot. Estimates of the effect sizes of all SNPs using inverse-variance weighted and MR-Egger analysis. (B) Results of various MR analysis methods. (C) Scatter plot. Results of Mendelian randomization analysis. (D) Forest plot. Leave-one-out analysis results for each SNP. MR = Mendelian randomization, SNP = single-nucleotide polymorphism, OR = odds ratio, IVW = inverse-variance weighted.

SNPs associated with AS were filtered based on a significance threshold (*P* < 5 × 10^−8^) and LD clumping (*R*^2^ <0.001, window size = 10,000 kb) as well as the *F*-statistic (*F* >10). These SNPs were subsequently merged with SNPs related to lung cancer, resulting in 35 eligible SNPs being used as IVs (Fig. 2A). Using 5 methods, including the IVW approach in MR analysis, we identified a significant potential causal relationship between AS and a reduced risk of lung cancer (*P*_IVW_ = 0.01, OR = 0.896, 95% CI = 0.825–0.974) (Fig. 2B and C). The MR-Egger intercept test implied the absence of horizontal pleiotropy in the MR analysis (*P* = .626). Furthermore, the leave-one-out sensitivity analysis demonstrated that no single SNP unduly influenced the stability of the overall results (Fig. 2D).

### 3.2. Identification of overlapping differentially expressed genes

**Figure 3.**
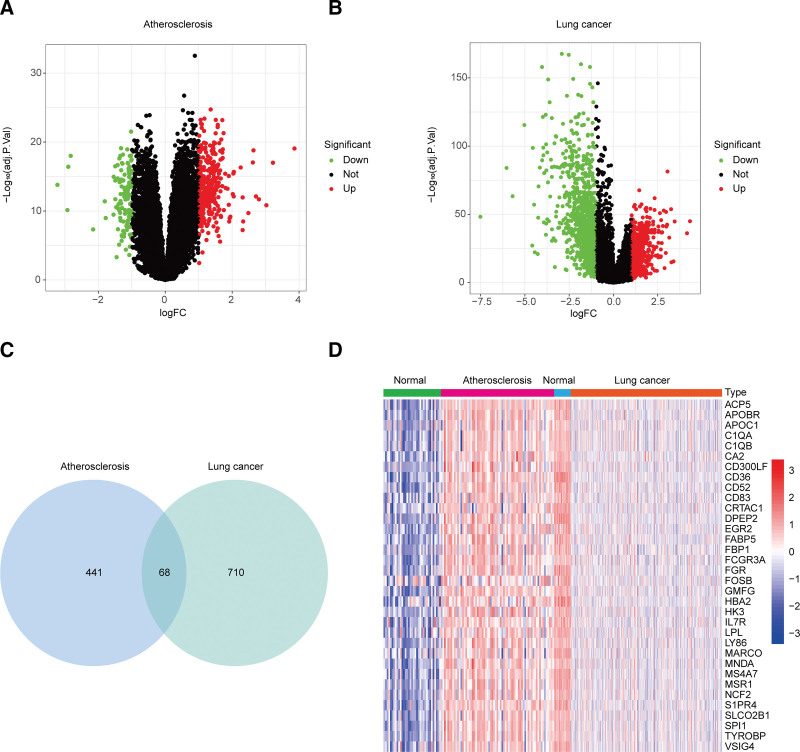
Screening of differentially expressed genes in AS and lung cancer. (A and B) Volcano plots of DEGs in AS and lung cancer. Red indicates upregulation, green indicates downregulation, and black indicates no significant change. (C) Venn diagram of intersecting genes between AS and lung cancer. (D) Heatmap of 34 DEGs. The horizontal axis represents sample groups, the vertical axis represents gene names, red indicates upregulation, blue indicates downregulation, and color intensity corresponds to the absolute value of logFC. DEGs = differentially expressed genes, logFC = log_2_ fold change, adj.*P* = adjusted *P*-value.

Following the normalization of microarray data, 441 and 710 DEGs were screened out in GSE100927 and the TCGA dataset, respectively (Fig. 3A). Among these, 68 DEGs were found to be differentially expressed in both AS and lung cancer (Fig. 3B and C). Notably, 34 DEGs that were hyperexpressed in AS and hypoexpressed in lung cancer were shown in a heatmap (Fig. 3D).

### 3.3. Enrichment analysis

**Figure 4.**
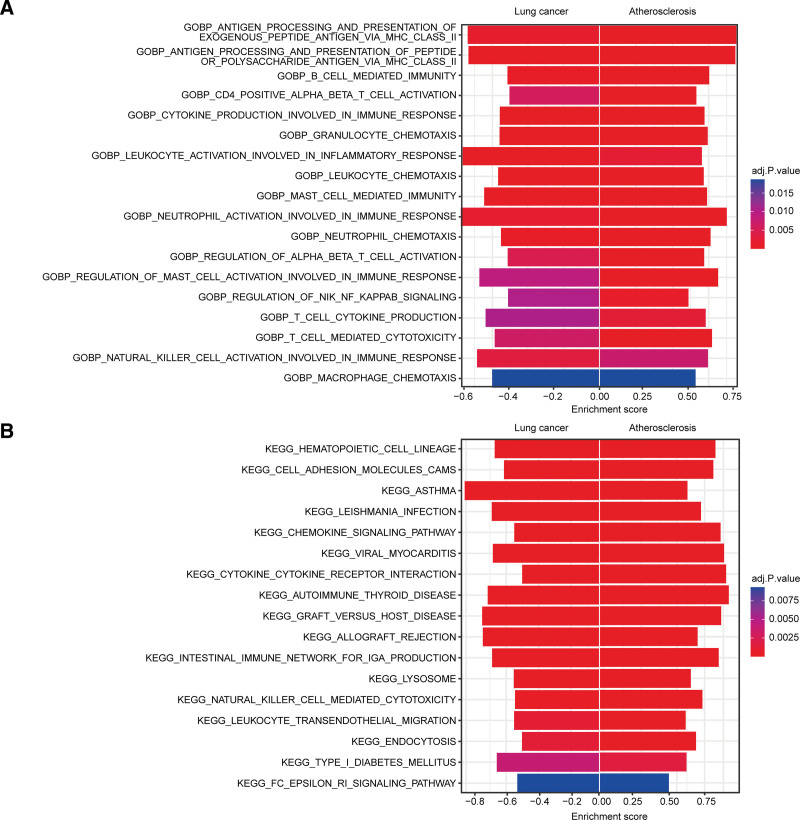
Results of GSEA enrichment analysis. (A) GO pathways altered in AS and lung cancer. (B) KEGG pathways are altered in both diseases. (A and B) The horizontal axis represents sample types, the vertical axis displays significantly enriched GO biological process entries, with colors corresponding to adj.*P*-values and bar lengths reflecting enrichment scores. adj.*P*-value = adjusted *P* value, GO = gene ontology, KEGG = Kyoto encyclopedia of genes and genomes.

GO and KEGG enrichment analyses were performed to investigate the potential functional roles of DEGs in relation to AS and lung cancer. We employed GSEA enrichment analysis to identify the principal signaling pathways implicated in the progression of both AS and lung cancer. An intersection analysis was subsequently conducted on these significant pathways (Fig. 4A and B). The results demonstrated that the majority of the discovered pathways were substantially associated with immunological functions, encompassing essential mechanisms such as immune response, immune cell activation, chemotaxis and migration, and regulation of inflammatory cytokines. The enrichment scores for AS-related pathways were predominantly positive, while those for lung cancer-related pathways were largely negative. In conclusion, a significant negative correlation existed between the activation of these pathways in the 2 illnesses.

### 3.4. Construction and validation of the recurrent-prognostic model

**Figure 5.**
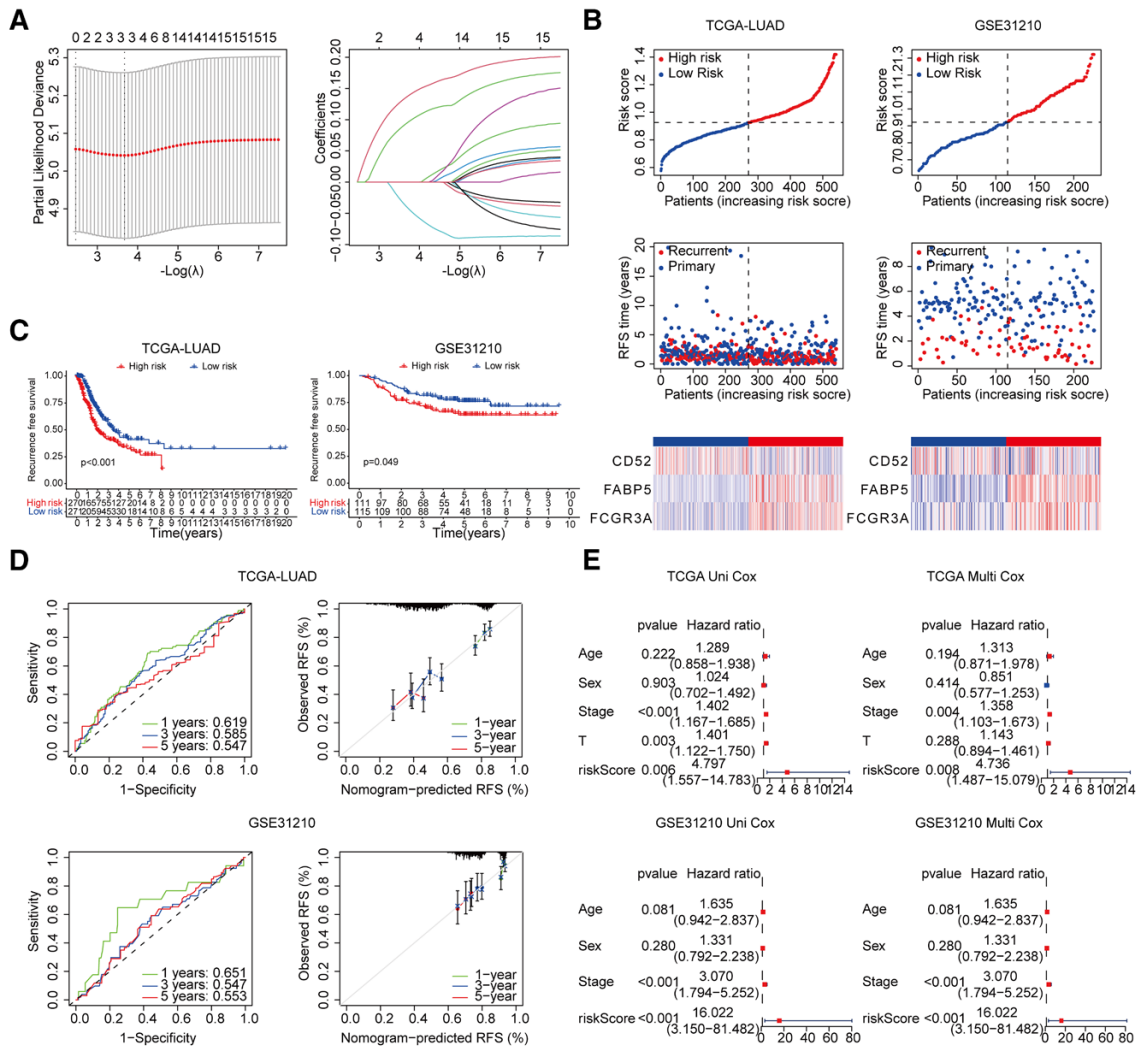
Construction and validation of a recurrence-related risk model for lung cancer in TCGA and GSE31210. (A) LASSO-Cox regression identified 3 key genes for model construction. Left: partial-likelihood bias versus penalty coefficient λ; right: coefficient paths colored by gene, with vertical line at optimal λ indicating selection. (B) The risk model stratified patients into low- and high-risk groups, with risk score distributions. Top: risk-score curves (patients ranked). Middle: survival status scatter. Bottom: heatmap of 3 core genes (red = high, blue = low). (C) K–M survival analysis showed the different distribution of RFS in low- and high-risk groups. The RFS curves demonstrate the survival disparity between the high-risk (red curve) and low-risk (blue curve) groups. (D) ROC curves and their calibration curves in TCGA and GSE31210 cohorts. Top: ROC curves for 1-, 3-, 5-year RFS prediction; Bottom: calibration plots (x-axis predicted vs y-axis observed probability). (E) Uni- and Multivariate Cox regression analysis confirmed that the risk scores were an independent prognostic factor for patients. The curve labeled “riskScore” represents the prognostic risk score constructed based on core genes; Cox regression refers to the proportional hazards regression model. TCGA-LUAD = the cancer genome atlas-lung adenocarcinoma, GSE31210 = gene expression omnibus, RFS = recurrence-free survival, ROC = receiver operating characteristic, Uni- = univariate; Multi- = multivariate.

Based on the MR and enrichment analysis results from the 68 common DEGs, the genes that were up-regulated in AS and down-regulated in lung cancer were selected. DEGs with |log2FC| >1.5 in both diseases were retained. Subsequently, 15 candidate DEGs were obtained. Following LASSO regression analysis, 3 predictive genes (CD52, FABP5, and FCGR3A) were ultimately identified for constructing the risk score (Fig. 5A). The risk score was calculated as follows: Risk score = −0.039 × Exp CD52 + 0.136 × Exp FABP5 + 0.094 × Exp FCGR3A. Based on the corresponding lambda value, the recurrence risk score for each patient was computed in both the TCGA dataset (training set) and the GEO dataset (validation set). Samples were then stratified into low- and high-risk groups (Fig. 5B). Patients in the high-risk group exhibited a higher incidence of death events and shorter overall survival times in both cohorts. Similarly, Kaplan–Meier survival curves demonstrated that patients in the high-risk group had significantly worse RFS (Fig. 5C). ROC curve analysis also indicated favorable predictive performance of the classifier (Fig. 5D). Furthermore, univariate and multivariate Cox regression analysis confirmed that the recurrence risk score model was an independent prognostic factor (Fig. 5E).

### 3.5. Low-risk patients showed higher immune cell infiltration levels

**Figure 6.**
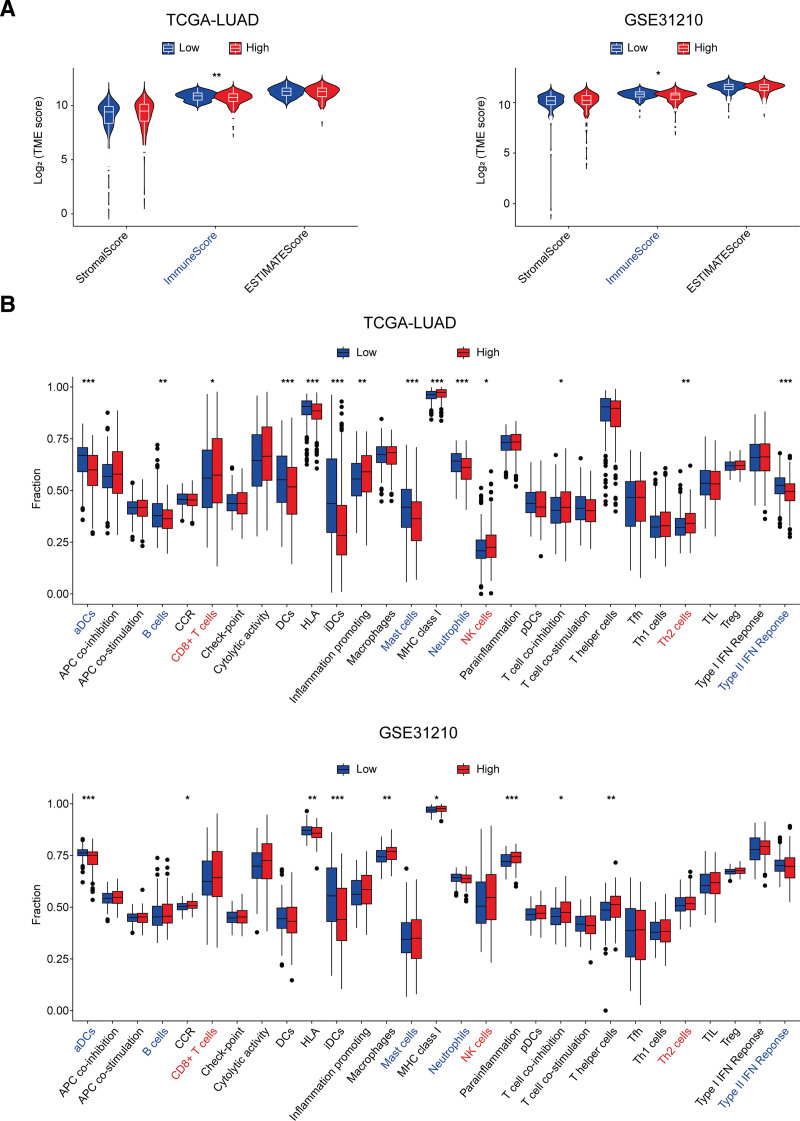
Results of tumor microenvironment analysis. (A) ESTIMATE analysis indicated that immune cells were more infiltrated in the low-risk group than in the high-risk group. The x-axis showed the risk classification type, and the y-axis displays the log-transformed risk score. StromalScore = stromal content; ImmuneScore = immune infiltration; ESTIMATEScore = combined TME index; Fraction = relative immune proportion. Low/high: risk groups. (B) The ssGSEA algorithm deconvoluted 29 types of immune cells/processes between risk groups. The x-axis represents immune cell/functional types (red = high, blue = low), and the y-axis shows the relative infiltration proportion of cells. ESTIMATE = estimation of stromal and immune cells in malignant tumor tissues using expression data, TCGA-LUAD = The cancer genome atlas-lung adenocarcinoma cohort; GSE31210 = validated lung adenocarcinoma dataset from the gene expression omnibus database, TME = tumor microenvironment, ssGSEA = single-sample gene set enrichment analysis.

The ESTIMATE results revealed that the low-risk group exhibited significantly elevated immune scores compared to the high-risk group (Fig. 6A), suggesting a more immunologically active state in low-risk patients. Subsequently, we applied ssGSEA to evaluate 29 immune cells or immune processes between the 2 groups. The analysis demonstrated that the low-risk group had markedly higher infiltration levels of dendritic cells, B cells, mast cells, and neutrophils. In addition, the type II interferon response has been significantly more activated in the low-risk group. In contrast, the high-risk patients showed significantly increased infiltration of CD8^+^ T cells, NK cells, and Th2 cells. These findings reflect distinct patterns of immune cell infiltration and functional states between the 2 groups. The low-risk group exhibited enhanced efficiency in antigen recognition, presentation, and immune activation, which may contribute to effective immune surveillance and tumor clearance. Furthermore, the immune microenvironment in the high-risk group appeared to be less conducive to tumor control and may facilitate immune evasion and poorer clinical outcomes (Fig. 6B).

## 4. Discussion

Lung cancer and atherosclerotic cardiovascular disease (ASCVD) remain the leading causes of death worldwide. In 2022, approximately 2.5 million new lung cancer cases were diagnosed globally, accounting for 12.4% of all cancer cases.^[19]^ Meanwhile, the number of prevalent cardiovascular disease cases worldwide increased from 271 million in 1990 to 523 million in 2019.^[20]^ Given the high prevalence of AS and lung cancer in the population and their frequent co-occurrence, considerable efforts have been devoted to exploring their association. However, no consensus has been reached so far.^[8,21]^ The MR analysis revealed an inverse association between AS and lung cancer risk, echoing a recent mouse-model study by Klement et al.^[10]^ Although the MR-Egger intercept test was nonsignificant (*P* = .143), its direction fully concurred with the IVW estimate, a pattern previously considered supportive in large-scale MR analyses with low-average-pleiotropy instruments [Ping W, Front Genet 2022; Liu H Front Genet 2023].^[22,23]^ We therefore regard the MR results as reliable genetic evidence for the relationship and potential mechanisms between the 2 diseases. The pathophysiology of AS and lung cancer involves multiple pathways; identifying novel molecules is key to dissecting their mechanisms,^[24,25]^ so we used transcriptomics to explore their interplay. Through the analysis of RNA-seq data from AS and lung cancer patients, 68 intersecting DEGs were identified. Among these, 34 genes exhibited upregulation in AS and downregulation in lung cancer. From this subset, we selected 3 key genes (CD52, FABP5, and FCGR3A) to construct an AS-related risk-prediction model, suggesting that these genes and their signaling pathways may act as common pathogenic factors for both diseases. Moreover, this inverse expression pattern provides molecular evidence supporting the MR conclusion that AS reduces lung cancer risk.

*CD52*, a glycoprotein broadly expressed on immune cells, suppresses immune responses by inhibiting lipopolysaccharide-induced cytokine production.^[26,27]^ It dampens T-cell activity,thereby attenuating atherosclerosis, yet its overexpression predicts poor survival in lung-cancer patients, presumably via glycolysis and inflammation.^[28,29]^ FABP5 regulates lipid metabolism and cell growth and also acts as a pro-inflammatory mediator in macrophages.^[30]^ In atherosclerosis, FABP5 promotes cholesterol efflux, limiting plaque progression,^[31]^ whereas in lung cancer it inhibits apoptosis by up-regulating the lipogenic gene SCD and its transcription factors.^[32]^ FCGR3A, expressed on NK cells, neutrophils, and macrophages, mediates antibody-dependent cellular cytotoxicity (ADCC).^[33]^ It accelerates atherogenesis through ADCC,^[34]^ but higher FCGR3A-enriched NK-cell levels correlate with better survival in lung-cancer patients, suggesting that ADCC impedes tumor progression.^[35]^ However, studies of these 3 crucial genes in AS and lung cancer are still scarce, which needs more exploration.

From risk model validation in K-M and ROC analysis, the AS-related 3-gene risk-prediction model effectively stratifies lung cancer patients with distinct prognoses. ESTIMATE results showed that there was a significant difference in TME immune activity between low- and high-risk groups. In the low-risk group, patients showed prominent infiltration of dendritic cells, neutrophils, and mast cells. In contrast, the high-risk group exhibited predominant infiltration of natural killer (NK) cells, CD8 + T cells, and Th2 cells.

The distinct immune infiltration profiles in the low- and high-risk groups were closely associated with inflammatory responses. Neutrophils and mast cells are central players in inflammatory responses. Mast cells, which are prevalent in atherosclerotic plaques, release diverse cytokines and chemokines through degranulation, including histamine, leukotrienes, TNF-α, and IL-6. These molecules exacerbate inflammation and further attract multiple immune cells, including neutrophils.^[36,37]^ Neutrophils are recruited to atherosclerotic inflammatory sites through chemotaxis. They release reactive oxygen species, myeloperoxidase (MPO), and other factors, contributing to plaque instability and rupture.^[38]^ Dendritic cells are activated by recognizing atherosclerosis-related antigens like oxidized low-density lipoprotein (ox-LDL). This activation initiates adaptive immunity and induces the release of pro-inflammatory cytokines, amplifying the local inflammatory response.^[36,39]^ In the tumor microenvironment, these cells similarly inhibit lung cancer development. Dendritic cells capture tumor antigens to initiate adaptive immunity against lung cancer. Mast cells release cytotoxic substances or mediate ADCC to suppress lung cancer cells. Tumor-associated neutrophils with the N1 phenotype directly kill tumor cells and induce apoptosis by releasing MPO and reactive oxygen species.^[40,41]^

In the tumor microenvironment, the antitumor functions of NK cells and CD8 + T cells are often compromised. For instance, NK cells infiltrating the lung cancer TME exhibit diminished cytotoxicity.^[42]^ Moreover, CD8 + T cells, subjected to chronic antigenic stimulation within the TME, become exhausted, showing reduced proliferation and cytotoxicity.^[43]^ Notably, Th2 cells in the TME release immunosuppressive cytokines like IL-10 and IL-4. IL-10 directly inhibits antitumor responses, while IL-4 promotes pro-tumorigenic M2 polarization of macrophages.^[44,45]^ Therefore, we speculated that immune regulatory pathways, which involve mast cells and neutrophils releasing cytokines and chemokines, recruit various immune cells and amplify inflammatory responses. Simultaneously, dendritic cells initiate adaptive immunity by recognizing specific antigens. This dual process exacerbates AS while suppressing lung cancer progression.

Chronic inflammation serves as a core characteristic in the pathogenesis of both AS and lung cancer.^[46,47]^ Immune cell infiltration into arterial walls promotes plaque formation and progression, acting as a central driver in atherosclerosis development. This process impairs vascular endothelial function, increases thrombosis risk, and accelerates cardiovascular disease (CVD) progression.^[46]^ While chronic inflammation is widely recognized as a cancer driver, recent studies have identified antitumor immune roles of specific inflammatory factors.^[48]^ For instance, IL-2 binding to receptors stimulates T-cell differentiation and reactivates depleted CD8 + T cells, thereby exerting cytotoxic effects to inhibit tumor progression.^[49]^ IL-33 inhibits tumor growth by promoting the antitumor activity of CD8 + T cells.^[50]^ We hypothesize that the 2 diseases may be interconnected through chronic inflammation, with AS potentially exerting an inhibitory effect on lung cancer development via immune molecules and related pathways. However, the specific mechanisms underlying these processes remain incompletely understood and warrant further investigation.

In conclusion, integrating MR and transcriptomic analyses, we demonstrated that AS is causally and negatively associated with lung cancer risk. Mechanistically, the chronic inflammatory milieu that drives AS plaque formation simultaneously restrains tumor development by mobilizing AS-associated immune cells-particularly neutrophils and mast cells-to secrete cytokines and activate adaptive immunity. Additionally, our risk-prediction model effectively stratified lung-cancer patients into high- and low-risk groups with distinct immune profiles, thereby linking AS-derived immune signatures to clinical outcomes.

It is imperative to underscore the limitations of this study. First, although MR analysis effectively mitigates potential biases inherent in observational studies, residual confounding factors remain difficult to fully eliminate. Second, the MR-Egger intercept was nonsignificant (*P* = .143), indicating low power to detect modest directional pleiotropy; however, as discussed, this does not invalidate the main IVW estimate. Finally, the current findings focus on 3 key genes (*CD52*, *FABP5*, and *FCGR3A*), and validation in larger, more heterogeneous cohorts is required to establish their generalizability.

## Author contributions

**Conceptualization:** Xike Wu.

**Data curation:** Peinan Zhang, Xiaozhang Bao, Xike Wu, Yuheng Yang.

**Formal analysis:** Xiaozhang Bao.

**Funding acquisition:** Nan Tian.

**Methodology:** Peinan Zhang.

**Resources:** Peinan Zhang.

**Supervision:** Xiaozhang Bao, Nan Tian.

**Validation:** Xiaozhang Bao.

**Visualization:** Xiaozhang Bao, Yuheng Yang, Yanqi Sun.

**Writing – original draft:** Peinan Zhang, Ying Qian, Nan Tian.

**Writing – review & editing:** Peinan Zhang, Ying Qian, Nan Tian.

## References

[1] Zhu Y Xian X Wang Z. Research progress on the relationship between atherosclerosis and inflammation. Biomolecules. 2018;8:80. 30142970 10.3390/biom8030080 PMC6163673

[2] Spagnoli LG Mauriello A Sangiorgi G. Extracranial thrombotically active carotid plaque as a risk factor for ischemic stroke. JAMA. 2004;292:1845–52. 15494582 10.1001/jama.292.15.1845

[3] Roth GA Johnson C Abajobir A. Global, regional, and national burden of cardiovascular diseases for 10 causes, 1990 to 2015. J Am Coll Cardiol. 2017;70:1–25. 28527533 10.1016/j.jacc.2017.04.052 PMC5491406

[4] Tapia-Vieyra JV Delgado-Coello B Mas-Oliva J. Atherosclerosis and cancer; a resemblance with far-reaching implications. Arch Med Res. 2017;48:12–26. 28577865 10.1016/j.arcmed.2017.03.005

[5] Katsi V Papakonstantinou I Tsioufis K. Atherosclerosis, diabetes mellitus, and cancer: common epidemiology, shared mechanisms, and future management. Int J Mol Sci. 2023;24:11786. 37511551 10.3390/ijms241411786 PMC10381022

[6] Gallucci G Turazza FM Inno A. Atherosclerosis and the bidirectional relationship between cancer and cardiovascular disease: from bench to bedside-part 1. Int J Mol Sci. 2024;25:4232. 38673815 10.3390/ijms25084232 PMC11049833

[7] Li C Lei S Ding L. Global burden and trends of lung cancer incidence and mortality. Chin Med J (Engl). 2023;136:1583–90. 37027426 10.1097/CM9.0000000000002529 PMC10325747

[8] Dreyer L Prescott E Gyntelberg F. Association between atherosclerosis and female lung cancer--a Danish cohort study. Lung Cancer. 2003;42:247–54. 14644511 10.1016/s0169-5002(03)00295-2

[9] Raposeiras Roubín S Cordero A. The two-way relationship between cancer and atherosclerosis. Rev Esp Cardiol (Engl Ed). 2019;72:487–94. 31053376 10.1016/j.rec.2018.12.010

[10] Klement H St Croix B Milsom C. Atherosclerosis and vascular aging as modifiers of tumor progression, angiogenesis, and responsiveness to therapy. Am J Pathol. 2007;171:1342–51. 17823292 10.2353/ajpath.2007.070298 PMC1988883

[11] Matetic A Mohamed M Miller RJH. Impact of cancer diagnosis on causes and outcomes of 5.9 million US patients with cardiovascular admissions. Int J Cardiol. 2021;341:76–83. 34333019 10.1016/j.ijcard.2021.07.054

[12] Ridker PM MacFadyen JG Thuren T Everett BM Libby P Glynn RJ; CANTOS Trial Group. Effect of interleukin-1β inhibition with canakinumab on incident lung cancer in patients with atherosclerosis: exploratory results from a randomised, double-blind, placebo-controlled trial. Lancet. 2017;390:1833–42. 28855077 10.1016/S0140-6736(17)32247-X

[13] Handy CE Quispe R Pinto X. Synergistic opportunities in the interplay between cancer screening and cardiovascular disease risk assessment: together we are stronger. Circulation. 2018;138:727–34. 30359131 10.1161/CIRCULATIONAHA.118.035516

[14] Lewis DR Pickle LW Zhu L. Recent spatiotemporal patterns of US lung cancer by histologic type. Front Public Health. 2017;5:82. 28580352 10.3389/fpubh.2017.00082 PMC5437205

[15] Huang Y Shi W He Q Tan J Tong J Yu B. Racial and ethnic influences on carotid atherosclerosis: epidemiology and risk factors. SAGE Open Med. 2024;12:20503121241261840. 39045542 10.1177/20503121241261840 PMC11265241

[16] Lovegrove CE Howles SA Furniss D Holmes MV. Causal inference in health and disease: a review of the principles and applications of Mendelian randomization. J Bone Miner Res. 2024;39:1539–52. 39167758 10.1093/jbmr/zjae136 PMC11523132

[17] Zhou W Nielsen JB Fritsche LG. Efficiently controlling for case-control imbalance and sample relatedness in large-scale genetic association studies. Nat Genet. 2018;50:1335–41. 30104761 10.1038/s41588-018-0184-y PMC6119127

[18] McKay JD Hung RJ Han Y; SpiroMeta Consortium. Large-scale association analysis identifies new lung cancer susceptibility loci and heterogeneity in genetic susceptibility across histological subtypes. Nat Genet. 2017;49:1126–32. 28604730 10.1038/ng.3892 PMC5510465

[19] Sung H Ferlay J Siegel RL. Global cancer statistics 2020: GLOBOCAN estimates of incidence and mortality worldwide for 36 cancers in 185 countries. CA Cancer J Clin. 2021;71:209–49. 33538338 10.3322/caac.21660

[20] Nedkoff L Briffa T Zemedikun D Herrington S Wright FL. Global trends in atherosclerotic cardiovascular disease. Clin Ther. 2023;45:1087–91. 37914585 10.1016/j.clinthera.2023.09.020

[21] Florido R Daya NR Ndumele CE. Cardiovascular disease risk among cancer survivors: the atherosclerosis risk in communities (ARIC) study. J Am Coll Cardiol. 2022;80:22–32. 35772913 10.1016/j.jacc.2022.04.042 PMC9638987

[22] Ping W Zhao Q Ge S Wang X Li F Huang X. Evaluating the effect of tanning response to sun exposure on the risk of skin diseases through Mendelian randomization. Front Genet. 2022;13:967696. 36118883 10.3389/fgene.2022.967696 PMC9478173

[23] Liu H Sun Q Bi W Mu X Li Y Hu M. Genetic association of hypertension and several other metabolic disorders with Bell’s palsy. Front Genet. 2023;14:1077438. 37533435 10.3389/fgene.2023.1077438 PMC10391645

[24] Pan H Ho SE Xue C. Atherosclerosis is a smooth muscle cell-driven tumor-like disease. Circulation. 2024;149:1885–98. 38686559 10.1161/CIRCULATIONAHA.123.067587 PMC11164647

[25] Tang M Yin Y Wang W. Exploring the multifaceted effects of Interleukin-1 in lung cancer: from tumor development to immune modulation. Life Sci. 2024;342:122539. 38423172 10.1016/j.lfs.2024.122539

[26] Ambrose LR Morel AS Warrens AN. Neutrophils express CD52 and exhibit complement-mediated lysis in the presence of alemtuzumab. Blood. 2009;114:3052–5. 19638623 10.1182/blood-2009-02-203075

[27] Rashidi M Bandala-Sanchez E Lawlor KE. CD52 inhibits Toll-like receptor activation of NF-κB and triggers apoptosis to suppress inflammation. Cell Death Differ. 2018;25:392–405. 29244050 10.1038/cdd.2017.173 PMC5762852

[28] Cai Y Zhao J Luo C. CD52 knockdown inhibits aerobic glycolysis and malignant behavior of NSCLC cells through AKT signaling pathway. J Cancer. 2024;15:3394–405. 38817869 10.7150/jca.86511 PMC11134428

[29] Zhang Y Liu Y Xie Z. Inhibition of PFKFB Preserves intestinal barrier function in sepsis by inhibiting NLRP3/GSDMD. Oxid Med Cell Longev. 2022;2022:8704016. 36589684 10.1155/2022/8704016 PMC9803577

[30] Hou Y Wei D Bossila EA. FABP5 deficiency impaired macrophage inflammation by regulating AMPK/NF-κB signaling pathway. J Immunol. 2022;209:2181–91. 36426981 10.4049/jimmunol.2200182

[31] Furuhashi M Ogura M Matsumoto M. Serum FABP5 concentration is a potential biomarker for residual risk of atherosclerosis in relation to cholesterol efflux from macrophages. Sci Rep. 2017;7:217. 28303004 10.1038/s41598-017-00177-w PMC5427929

[32] Wang Q Zhou J Cheng A. Artesunate-binding FABP5 promotes apoptosis in lung cancer cells via the PPARγ-SCD pathway. Int Immunopharmacol. 2024;143:113381. 39405934 10.1016/j.intimp.2024.113381

[33] Oboshi W Watanabe T Yukimasa N. SNPs rs4656317 and rs12071048 located within an enhancer in FCGR3A are in strong linkage disequilibrium with rs396991 and influence NK cell-mediated ADCC by transcriptional regulation. Hum Immunol. 2016;77:997–1003. 27338556 10.1016/j.humimm.2016.06.012

[34] Zhang Q Liu H Zhang M Liu F Liu T. Identification of co-expressed central genes and transcription factors in atherosclerosis-related intracranial aneurysm. Front Neurol. 2023;14:1055456. 36937519 10.3389/fneur.2023.1055456 PMC10017537

[35] Ru K Cui L Wu C. Exploring the molecular and immune landscape of cellular senescence in lung adenocarcinoma. Front Immunol. 2024;15:1347770. 39267750 10.3389/fimmu.2024.1347770 PMC11390420

[36] Guo X Sun M Yang P Meng X Liu R. Role of mast cells activation in the tumor immune microenvironment and immunotherapy of cancers. Eur J Pharmacol. 2023;960:176103. 37852570 10.1016/j.ejphar.2023.176103

[37] Galli SJ Gaudenzio N Tsai M. Mast cells in inflammation and disease: recent progress and ongoing concerns. Annu Rev Immunol. 2020;38:49–77. 32340580 10.1146/annurev-immunol-071719-094903

[38] Zhang X Kang Z Yin D Gao J. Role of neutrophils in different stages of atherosclerosis. Innate Immun. 2023;29:97–109. 37491844 10.1177/17534259231189195 PMC10468622

[39] Qian C Cao X. Dendritic cells in the regulation of immunity and inflammation. Semin Immunol. 2018;35:3–11. 29242034 10.1016/j.smim.2017.12.002

[40] Masucci MT Minopoli M Carriero MV. Tumor associated neutrophils. their role in tumorigenesis, metastasis, prognosis and therapy. Front Oncol. 2019;9:1146. 31799175 10.3389/fonc.2019.01146 PMC6874146

[41] Sheervalilou M Ghanei M Arabfard M. Tumor-asso7ciated neutrophils and neutrophil extracellular traps in lung cancer: antitumor/protumor insights and therapeutic implications. Med Oncol. 2025;42:266. 40522596 10.1007/s12032-025-02831-0

[42] Russick J Torset C Sun D. Tumor stage-driven disruption of NK cell maturation in human and murine tumors. iScience. 2024;27:111233. 39583926 10.1016/j.isci.2024.111233 PMC11585790

[43] Zhang B Liu J Mo Y Zhang K Huang B Shang D. CD8^+^ T cell exhaustion and its regulatory mechanisms in the tumor microenvironment: key to the success of immunotherapy. Front Immunol. 2024;15:1476904. 39372416 10.3389/fimmu.2024.1476904 PMC11452849

[44] Hanahan D Michielin O Pittet MJ. Convergent inducers and effectors of T cell paralysis in the tumour microenvironment. Nat Rev Cancer. 2025;25:41–58. 39448877 10.1038/s41568-024-00761-z

[45] DeNardo DG Barreto JB Andreu P. CD4(+) T cells regulate pulmonary metastasis of mammary carcinomas by enhancing protumor properties of macrophages. Cancer Cell. 2009;16:91–102. 19647220 10.1016/j.ccr.2009.06.018 PMC2778576

[46] Galkina E Ley K. Immune and inflammatory mechanisms of atherosclerosis (*). Annu Rev Immunol. 2009;27:165–97. 19302038 10.1146/annurev.immunol.021908.132620 PMC2734407

[47] O’Callaghan DS O’Donnell D O’Connell F O’Byrne KJ. The role of inflammation in the pathogenesis of non-small cell lung cancer. J Thorac Oncol. 2010;5:2024–36. 21155185 10.1097/jto.0b013e3181f387e4

[48] Haabeth OA Bogen B Corthay A. A model for cancer-suppressive inflammation. Oncoimmunology. 2012;1:1146–55. 23170261 10.4161/onci.21542 PMC3494627

[49] Shouse AN LaPorte KM Malek TR. Interleukin-2 signaling in the regulation of T cell biology in autoimmunity and cancer. Immunity. 2024;57:414–28. 38479359 10.1016/j.immuni.2024.02.001 PMC11126276

[50] Okuyama Y Okajima A Sakamoto N. IL-33-ILC2 axis promotes anti-tumor CD8^+^ T cell responses via OX40 signaling. Biochem Biophys Res Commun. 2022;637:9–16. 36375254 10.1016/j.bbrc.2022.11.006

